# Moral Awareness and Animal Welfare

**DOI:** 10.1017/awf.2023.33

**Published:** 2023-05-15

**Authors:** Heather Browning, Walter Veit

**Affiliations:** 1 Department of Philosophy, University of Southampton, UK; 2 Department of Philosophy, University of Bristol, UK

As philosophers working in both animal welfare science and animal ethics, we were happy for the chance to review this book. Despite the importance of animal welfare science for animal ethics, few philosophers and ethicists have made the effort to closely engage with this branch of science, and Lamb’s book provides an opportunity for people interested in bringing ethics and animal welfare science closer together. The book is accessibly written and though it is intended for a broad audience, it mostly targets animal welfare researchers who have less familiarity with the philosophical and animal ethics literature.

This is an expansive book covering an eclectic range of subject matter, and the author often detours from the core topics of his book. He freely admits to pursuing his lines of thought wherever they may lead, giving rise to frequent digressions and side topics; all relevant in some way or another to the broader subject at hand, but not arranged in a systematic manner. The discussion includes a lot of examples from different contexts, such as keeping animals in zoos, eating dogs, use of electric shock training aids, pedigree dog breeding, and the development and application of welfare legislation, showing that Lamb has thought a lot about these issues and their application.

The book is highly opinionated, and most readers will find much to both agree and disagree with. He covers a range of controversial topics, such as the role of anthropomorphism and anti-anthropomorphism in the animal sciences, the relevance of autonomy to moral consideration, the possession of concepts and use of language by animals, the role of animal aesthetics, the importance of caring relationships with animals, and the harms of killing animals. However, the primary focus is on two separate but related topics – the relevance of consciousness to moral status, and the scientific investigation of consciousness.

In his discussion on animal ethics, he surveys prominent ethical theories, arguing against the common idea that moral concern for animals should rest on the possession of empirically detectable properties such as sentience, rationality, or personhood. While we agree that use of such properties requires careful justification, this is not the same as a rejection of them entirely. For instance, he pushes back on the commonly held idea that sentience is an appropriate criterion for moral consideration, taking it to be ‘arbitrary’; though most who support this position would not agree with this characterisation, having principled reasons for adopting the stance. However, his point that too much focus on empirical capacities may undermine our direct relationships with animals is a sound one.

He is clearly and obviously anti-utilitarian, and this background influences his arguments throughout, in which opposing viewpoints are often presented uncharitably. The role of utilitarian thinkers such as Bentham and Singer who were hugely influential in expanding moral consideration to animals, is unfortunately downplayed. Some of the discussion of pleasures and pains are shallow, with little reference to the current science of affect. There is a strong emphasis on the role of moral intuition in determining the best ethical principles, with a commitment to ‘universal values’ and, in particular, he seems to be drawn to a ‘virtue ethics’ approach for exploring the different context-dependent features that should guide moral decision-making. However, it is unclear how strongly we should rely on our moral emotions – after all, they did not evolve to care impartially for other animals (or for that matter, even other humans) and they have been frequently used to justify many kinds of harms. At times, readers who do not share his background intuitions may struggle to keep up with the direction of the discussion and despite taking up a large part of the book, we suspect that the arguments will not convince many who (like ourselves) already fall broadly within the utilitarian tradition.

He is critical of the view he attributes to animal welfare studies – that the empirical study of animal welfare can replace ethical discussion – providing a characterisation of welfare scientists as attempting to pursue an objective or value-free investigation into animal suffering based on narrow cost-benefit calculations. However, we disagree somewhat with this characterisation of what welfare scientists are trying to do. Rather than attempting to make ethical claims about how welfare should matter morally, we see welfare science as taking it as a starting point that welfare does matter in some way or another, and then working to find how to improve it for a range of animals living in different conditions. The investigation of what is good or bad for animal welfare leaves open what is to be done with this information. Similarly for questions of commensurability of interests – identifying or measuring the suffering of different beings does not require any commitment to the different ethical weights that might be placed upon them.

We do not see that his claim that this scientific output needs to be situated within further philosophical and ethical discussion would be one that is controversial to many. While he is right to recognise that science is not and cannot be as ‘value free’ as is often claimed, this is not the same as requiring scientists to also be ethicists. The fact that scientists choose to focus on the assessment of suffering does not have to imply that they think this is all that matters morally – rather it may be all that is within their scope to investigate. Most animal welfare scientists care greatly about and want their research to benefit animals, but that doesn’t mean that animal welfare isn’t a natural phenomenon that can’t be studied independently of this. Regardless of what one thinks about this, the questions regarding the interplay between science and ethics are important issues for any welfare scientist to think through for themselves, and this book provides many thought-provoking ideas to guide reflection.

He is also highly (and, we think, overly) sceptical of the science of animal consciousness, referring to attempts to make inferences about animal mental states as “philosophical nonsense.” Instead, he seems to prefer a form of direct ‘recognition’ of the sentience of other creatures, overlooking the fact that what seems from a first-person point of view to be a form of direct perception is almost certainly the result of complex unconscious inference, based on a range of cues interpreted through learning and previous experience. Even very simple perception is likely to involve a lot of construction and prediction by the brain. While access to another’s feelings or emotions may *feel* immediate to most, this is not evidence that it is not the result of many unconscious inferences based on behavioural and bodily cues. Indeed, the reported difficulties by some neurodivergent people with making such interpretations suggests that there is an underlying process most are simply unconscious of. The use of reasonable inference and background theory to support the scientific study of animal consciousness is left unexplored. Instead, it is taken as given that we as humans are able to immediately and accurately identify other conscious beings.

However, though we disagree with his take on the study of animal consciousness in general we did agree with his discussion on the use of ‘critical anthropomorphism: “a method of critically using human experiences to recognise elements of an animal’s mental states” (p. 162), carefully using relevant similarities based in evolutionary theory to ground inferences in order to avoid both over-attributing human-like mental states to animals, but also being needlessly sceptical about the possession of some mental states by animals. He is correct to note that in some parts of animal welfare science, scepticism about our ability to objectively study animal minds hampers progress on understanding animal feelings and for this reason these are considerations that welfare scientists and ethicists should be paying attention to.

Despite some of the disagreements described above, we were impressed by the range of topics covered by this book. There is discussion of many issues in animal ethics that will provide a lot of food for thought for people working (or living) with animals. The ethical debates are presented in a simple and accessible manner and could in principle be read by anyone, so long as they keep in mind that the author is providing this survey with a distinct point of view and should thus be read with a critical eye. The book raises many thought-provoking questions, and we believe it would make for a worthwhile library addition for those interested in the ethical questions that lie alongside the scientific study of animal welfare.

